# How regulatory orders and public fear affect vehicle mobility under COVID-19: A global perspective from urban overall vehicles using multi-source data

**DOI:** 10.1371/journal.pone.0325118

**Published:** 2025-06-11

**Authors:** Li Tang, Chuanli Tang, Hao Luo

**Affiliations:** 1 Vehicle Measurement, Control and Safety Key Laboratory of Sichuan Province, Xihua University, Chengdu, Sichuan, China; 2 School of Automobile and Transportation, Xihua University, Chengdu, Sichuan, China; Southwest Jiaotong University, CHINA

## Abstract

COVID-19 has had a significant impact on global transportation. While extensive research has focused on its influence on public transit and shared travel, the changes in overall vehicle travel demand remain under – explored. This paper analyzes the magnitude, duration, and driving factors of the impact of COVID-19 on urban overall vehicle mobility. We introduced the Prophet time series model, a suitable tool for time – series prediction in this context, to predict vehicle mobility without the pandemic. By comparing the predicted and real values, the mobility loss was derived. Multiple linear regression was then applied to deeply explore the causes of this loss, with a particular focus on the interaction effects of the strictness of regulatory orders and public fear. A large-scale dataset with over 4 billion raw data from multiple sources was used for empirical analysis. Results indicate that 29.66% of urban vehicle mobility loss occurs during the national outbreak period. The factor representing nationwide confirmed cases has a positive lagging effect on travel mobility loss, with a lag time of around seven days. The loss of urban motorized travel demand is largely due to the interaction of perceived risks and control policies.

## 1. Introduction

In December 2019, COVID-19 broke out and then spread rapidly to many countries around the world in early 2020, gradually becoming a global pandemic. The COVID-19 pandemic has significantly altered human mobility patterns, an essential component of urban functionality, as mobility enables people to access vital services such as employment, housing, healthcare, and recreation [1]. These mobility changes were driven not only by regulatory orders (e.g., city lockdowns, stay-at-home orders, and social distancing policies [2–7]), but also by subjective adjustments in individuals behavior due to public fear, as many people chose to stay at home or shifted to more private modes of transportation (such as walking, motorcycles, and private cars) to reduce the risk of virus transmission [8–11]. It is crucial to understand the dynamics of pandemic evolution and to quantify the effects of key factors like public fear and the strictness of regulatory orders. In particular, attention must be given to how these effects vary across different stages of the pandemic [12]. Such understanding is essential for policymakers to develop precise and cost-effective response strategies. Furthermore, the strategies and insights gained during the COVID-19 response can provide valuable guidance for managing and mitigating the impacts of future pandemics.

Abundant empirical evidence has shown that the prevalence of the pandemic significantly reduces people’s willingness to travel [13–16], especially using public transit [17–21] and travel and shared travel [22–25]. In the meanwhile, the contributions to travel demand drop of different driven factors have been largely discussed in the literature, such as voluntary change of behaviour or mandatory orders [26,27]. However, how and why the overall motorized travel (including both public and private) changes, have not been fully addressed. Therefore, our first research question is raised: (*RQ–1*) *how does a pandemic like COVID-19 make an impact on overall vehicle mobility as to the magnitude and duration?* Proper answers could reveal the fluctuation of urban residents’ motorized travel, while the impact of different variables on the overall vehicle travel willingness can be observed.

Moreover, most of the data used in existing research for analysis are either questionnaire data or order data provided by specific transportation service providers. Analysis based on these data can help us understand the factors that alter people’s travel modes (e.g., from public transit to private cars). However, it is difficult to capture the overall impact of the pandemic on human mobility at the city scale. This leads to our second research question: (*RQ–2*) *How can large-scale spatiotemporal data be mined to quantify the macro impact of the pandemic on urban transportation?*

To answer the above research questions (*RQ–1* and *RQ–2*), this paper explores the impact of COVID-19 from a global perspective of vehicle mobility. It contributes to the literature in two folds.

- It supplements the vastly growing body of COVID-19 studies with a novel perspective of overall vehicle mobility. A large-scale dataset with more than 4 billion raw data was built from three different sources, including a three-year city-level automatic number plate recognition (ANPR) data and data crawled from websites relating to explanatory variables. This provides a paradigm for how to investigate the impact of public health emergencies such as COVID-19 on travel behaviour through big data technology.
- The impact of regulatory orders and public fear is quantitively analyzed, particularly focusing on the inter-effect of these influencing factors. Caution fatigue, a psychological concept which refers to the decreased sensitivity or responsiveness to warnings, risks, or safety measures after prolonged exposure, is observed and confirmed [28–30]. The characteristics of caution fatigue under different influencing factors are explicitly analyzed. It will help relevant interests to make better decisions in responding to similar public health emergencies in the future.

The rest of the paper is organized as follows. Section 2 provides a review of existing studies on travel behaviour changes after COVID-19 and the main driving factors. Section 3 introduce the data and the modelling approach. Section 4 presents the calculation results of vehicle mobility loss and estimates of multiple linear regression as well as behavioral outputs of the empirical data. Section 5 provides a deeper discussion on factor selection, model construction, and application. Finally, Section 6 concludes the paper and points out future research directions.

## 2. Literature review

### 2.1. Travel loss/transition under COVID-19

A sharp decrease in mobility during the first wave of COVID-19 has been widely observed, even though the start time of the pandemic outbreak varied from country to country [5,31–35]. In addition, increasing evidence shows that people’s travel behaviour has also been altered. Multiple studies from major metropolitan areas across the world supported that there was a tendency for mode transition from public transit to private vehicles. Padmakumar and Patil [36] compared the spatiotemporal differences in travel mode usage during the national lockdown and unlock periods, using Google and Apple mobility data from six Indian cities. The result shows that individual travel modes (i.e., walking and driving) are preferred over public transport across all the cities in both periods. Liu et al [37] studied how urban travel behaviour changes along with the lockdown and the reopening of eight large cities in China based on the traffic congestion index and subway ridership data. The result shows that the values of the average travel time in the urban road network of the study area decreased to 54% −79% of the previous level, and the subway ridership dropped by more than 90% during the lockdown period. In the reopening period, vehicle traffic volume recovered much faster than the subway ridership, suggesting a shift of mode choice from public transit to private cars. Chen et al [26] reported that even without government intervention, railway ridership dropped by 40% to 60% during the pandemic peak in Taiwan, while roadway traffic increased by 20%.

### 2.2. Factors and social-demographic groups

Many factors may lead to changes in mobility and travel behaviour. Multiple studies have shown that executive stay-at-home orders could significantly reduce personal mobility [27,38]. This effect will be further reinforced by additional anti-COVID-19 policies, such as wearing masks, restricting traffic, and maintaining social distance controls [7,39,40,41]. Apart from regulatory orders with a mandatory nature, strong evidence provided by Chen et al [26] has indicated that people’s perception of risk would drive individuals to curtail their movements or substitute private for public transport to reduce exposure to the virus, even in an area without governmental restrictions. Shelat et al [18] conducted a stated choice experiment on train passengers in the Netherlands at the end of the first pandemic wave. The result shows that over two-thirds of travellers change their choices based on their perceived risks of virus transmission.

Interestingly, a portion of scholars have observed that factors affecting travel behaviour have also changed as the coronavirus continuously mutates and its toxicity varies. Kim and Kwan [13] used county-level smartphone data to study changes in people’s mobility by differentiating the COVID-19 pandemic in the U.S. into two waves. The study result reveals that restricting people’s mobility to control the pandemic may be effective only for the short term (in the second wave, very little mobility declined even though restriction policies still exist and the pandemic becoming more severe). Liu and Yamamoto [42] estimated inner and inter-city population mobility in the Tokyo Metropolitan Area using mobile data and found that the correlation between mobility and the spread of COVID-19 gradually weakens as time goes by.

Moreover, significant differences in travel behaviour under COVID-19 have been found across social-demographic groups. A substantial body of empirical research from multiple countries like Switzerland, Spain, Italy, Bangladesh, Australia, and India indicates that concerning the high infectivity of the virus, a major group of travellers has shifted their mode choices from traditional public transport to private travel modes (e.g., driving, walking, and cycling). The degree of this alternation is not evenly distributed among different social groups [32,36,38,43–46]. The study from Chicago shows that the proportion of white and Asian populations tends to be positively correlated with the reduction of passengers in the railway and bus systems, while the proportion of black people is negatively correlated [39]. The number of public transport passengers decreases more in areas with high-intensity commercial land use, white people, educated, and high-income groups [47]. Asians are less likely to walk than whites due to the double impacts of the virus and racial discrimination, based on a field investigation in Melbourne during the lockdown period [48]. People with low income significantly reduced their travel frequencies and they walked/cycled more according to research results from Australia and Turkey [49,50]. Men, freelancers, and less educated people tend to travel more frequently and are more loyal to public transit in Spain and Germany [32,51].

To summarize, COVID-19 has largely reduced people’s activity and travel (especially riding with public transit). It also causes a stronger negative impact on socially vulnerable groups (such as low-income, low-education, and the elderly). This mobility reduction is caused by, on the one hand, restriction policies that force limitation on travel, and on the other hand, by public fear. However, some important questions remain to be addressed. For example, how much is the mobility loss of urban vehicles during the study period? What are the main factors contributing to the loss? How do the effects of these factors vary over time during the different stages of the pandemic? What can the government and traffic departments learn from it in the post-pandemic era for a better response to the next crisis? This paper aims to bridge this research gap.

## 3. Materials and methods

### 3.1. Data preperation

This paper takes Mianyang in Sichuan province, China as the background of the empirical research. Mianyang is a medium-sized city with an area of 20,200 km^2^ and a population of 4.88 million. Considering that the research timeframe of this study ends in 2020, we use this year as the statistical reference for relevant socio-economic and geographical data. The gross domestic product (GDP) of Mianyang reached 301 billion RMB, making it the second-largest economy in Sichuan Province and one of China’s top 100 GDP cities [52]. The city administers three districts and five counties, with its central urban area covering 554 km^2^, a permanent population of 1.3 million, and a population density of approximately 2,348 people per km^2^. The GDP of the central urban area was 107 billion RMB in 2020. The total road length in the central urban area was 1,356 kilometers, with 200,554 registered private cars, resulting in a per capita car ownership of 0.15 vehicles per person [53]. The city had 197 public bus routes, with a 72% coverage rate within a 500-meter radius of bus stops. Public transportation accounted for 59% of motorized travel [54]. There are no subway, light rail, or tram systems in Mianyang, and although an inland river flows across the central urban area, the ferries operating there are mainly tourist boats and do not serve transportation purposes. For short-distance travel, as Mianyang is located in the Sichuan Plain, approximately 28,000 shared bicycles are deployed in the central urban area. Therefore, it can be concluded that buses are the primary mode of public transportation, while walking or cycling is the primary mode for short-distance travel.

Unlike Beijing, Chengdu, or other first-tier and second-tier supercities, Mianyang belongs to the middle city class or the so-called third-tier cities. However, according to the Seventh National Population Census, China has 278 cities with a similar administrative level and population economic scale as Mianyang, accounting for 40.6% of all cities. This makes the empirical study results representative. We believe that it is valuable for cities like Mianyang to observe the impact of COVID-19 on activity and travel from the perspective of vehicle mobility. In medium-sized cities, buses are typically the primary mode of public transport. This is mainly due to the strict approval requirements for the construction of rail transit in China. In the 337 cities across the country, only 59 have opened and operated urban rail lines, with the vast majority being mega and large cities [55]. Therefore, for most medium-sized cities like Mianyang, medium and long-distance travel is mostly taken by different types of vehicle transport including private cars, taxies, hailing cars and buses. It is worth noting that two-wheeled electric vehicles are also very popular in China. However, we are unable to obtain accurate data on their ownership and usage through official channels. Therefore, this paper does not include analysis or discussion related to two-wheeled electric vehicles.

**Fig 1:**
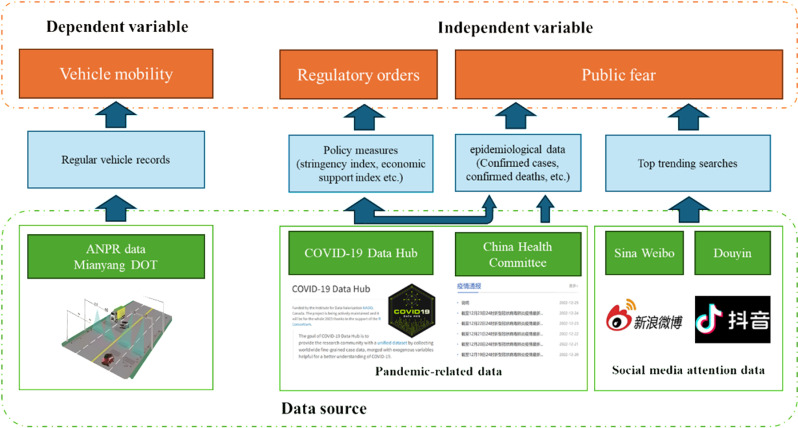
Multi-data sources and their relationships with the main causal variables.

The dataset was collected from three sources and used to form dependent and independent variables in our analysis model. Fig 1 illustrates the data sources and their relationships with the main causal variables.

The first and most essential data is the city-level ANPR data obtained from the Mianyang Department of Transportation. Major fields in the raw ANPR dataset include time stamps, vehicle license plate numbers, plate types, and device IDs with longitude and latitude information. The use of this data complies with all relevant terms, conditions, and data protection requirements established by the Mianyang Department of Transportation. Considering data availability and the research objective, the study period of this paper is defined as starting from January 21, 2018, to August 30, 2020. The dataset includes more than 4.3 billion records in total, with an average daily original record of about 4.4 million. The average number of available ANPR detectors is about 550 per day.

**Fig 2:**
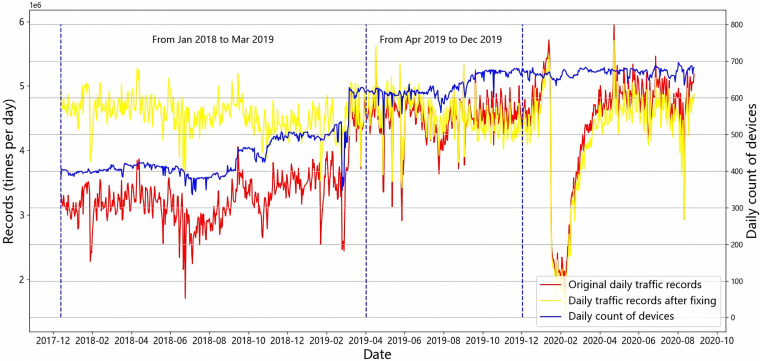
The trend of vehicle mobility before and after data fixing.

Handling such a vast amount of data over a long spatiotemporal span is a significant challenge. The processing work of ANPR data includes data cleaning, extraction, and fixing. To clean the raw data, duplicated data was dropped and invalid data (such as stained or unrecognized license plate data), abnormal data, and missing data were omitted. As our research focus is urban residents’ vehicle mobility, records belonging to regular travel vehicles (i.e., yellow card car, blue card car, hybrid car, and new energy car) were extracted out of eight types of plates. During the data cleaning process, we found it necessary to adjust the daily traffic records due to fluctuations in the number of operational ANPR detectors over the study period. Ideally, traffic counts would be extracted from a consistent group of ANPR devices. However, this was not feasible, as the number of active devices varied daily (as shown by the blue line in Fig 2), and sensor IDs frequently changed due to ongoing upgrades and modifications to the city’s ITS infrastructure. This inconsistency introduced systematic measurement errors in the raw daily records.

To correct for these biases, we applied a mathematical adjustment method, which covers the full study period from January 1, 2018 to August 30, 2020 and can be expressed as


$$\Delta rpd=\frac{{\overset{―}{r}}_{2}-{\overset{―}{r}}_{1}}{{\overset{―}{d}}_{2}-{\overset{―}{d}}_{1}}$$ (1)

$${\hat{r}}_{t}=\Delta rpd\times ({\overset{―}{d}}_{2}-d_{t})+r_{t}$$ (2)

Where Δrpd is defined as the average number of vehicles that can be captured by an ANPR sensor, with the assumption that the total traffic volume before COVID-19 in the study area remains steady; ${\overset{―}{r}}_{1}$ and ${\overset{―}{r}}_{2}$ denote the average daily numbers of records in Periods 1 and 2, while ${\overset{―}{d}}_{1}$ and ${\overset{―}{d}}_{2}$ denote the average daily numbers of available devices in the two periods; $d_{t}$ and $r_{t}$ denote the real number of available devices and the number of records on day t, respectively; ${\hat{r}}_{t}$ is the fixed number of records on day t. This adjustment allows us to mitigate the effects of infrastructure-related fluctuations and ensures that the resulting data more accurately reflects actual vehicle mobility trends.

Fig 2 shows that the fluctuation of the fixed traffic records (yellow line) from January 2018 to March 2019 is consistent with those from April 2019 to December 2019. The fixed records maintain the same tendency as the original data (red line) and become smoother, indicating that the fixing method works. Therefore, the fixed traffic records, defined as vehicle mobility (yellow line) in our paper, are used for the follow-up study.

The second data source is pandemic-related data. It is mainly obtained from a public dataset called COVID-19 Data Hub (https://covid19datahub.io), a Non-Profit Organization (NPO) funded by the Institute for Data Valorization IVADO, Canada [56]. The goal of COVID-19 Data Hub is to provide the research community with a unified dataset by collecting fine-grained case data, merged with exogenous variables helpful for a better understanding of COVID-19. Some supplementary data was collected from the official website of the local Health Committee in China. This dataset includes some regulatory order indicators and epidemiological variables (e.g., the cumulative number of confirmed cases, the cumulative number of deaths, total population, etc.). All data from these sources are publicly available and were used in accordance with their usage policies. Considering the research objectives and referring to relevant literature [39], we selected stringency index and economic support index as regulatory order indicators, where the former represents the strictness of “lockdown style” policies, and the latter measures such as income support and debt relief during the study period were selected.

**Fig 3:**
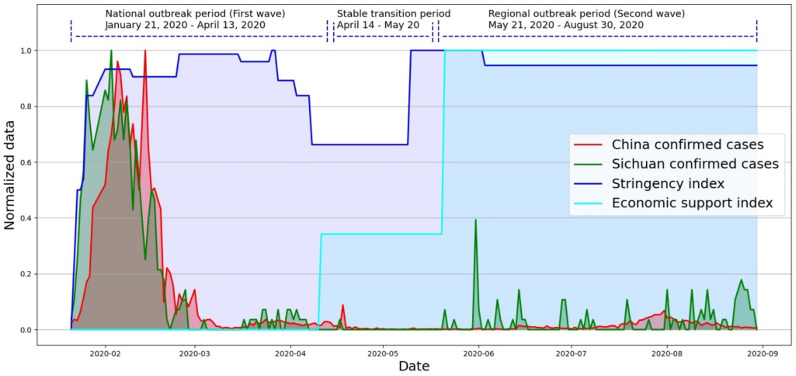
Visualization of the confirmed cases data variation with the changes of policy indexes.

Literature shows that people’s public fear during a pandemic could be measured based on some quantifiable indicators such as daily confirmed cases and daily deaths [57]. Additionally, some studies indicate a positive correlation between social media attention and perceived risk [58]. Fig 3 visualizes normalized confirmed cases data and the regulatory order data. Seeing from the confirmed cases (green line and red line), the trend of cases in Sichuan Province remains consistent with the overall situation of the country, but the former line reaches the peak before the latter. Since the outbreak of COVID-19, the strictness of policies (blue line) has rapidly strengthened and maintained at a high level all the time. Economy support (cyan line) started and increased gradually after the overall pandemic situation in the country was under control. Then, it remained at a high level even when the pandemic re-occurred in some local areas, indicating that the macro policy at that time still tended to minimize the impact of COVID-19 on economic development. Considering the abovementioned conditions, three distinct periods can be recognized from Fig 3. We named them as the national outbreak period (January 21, 2020 – April 13, 2020), stable transition period (April 14, 2020 – May 20, 2020), and regional outbreak period (May 21, 2020 – August 30, 2020) respectively.

The third dataset is the social media attention data, which is generated by the crawler result of the historical top trending searches from the two most popular social media (i.e., Sina Weibo and Douyin) in China. Sina Weibo is known as the Chinese version of Twitter. It accounts for 57% of China’s total microblog users and 87% of China’s total microblog activity. Douyin is a short video social app that can be viewed on smartphones. At the writing of this paper, the number of Douyin users reaches about 809 million, and the daily active number exceeded 700 million. 18,787 records were obtained from Sina Weibo and Douyin by utilizing requests and lxml packages of Python. These data were obtained using web scraping techniques that adhered to the platforms’ publicly available terms of service. No personal information was collected, and only aggregate trend data were used for analysis. This dataset is available from the corresponding author upon reasonable request.

This indicator together with the epidemiological variables from the second dataset, constitute the relevant variables representing public panic in our analysis model. Specifically, according to the relation to the pandemic, all the trend topics were divided into two categories, using keyword filtering and manual proofreading. The keywords used in the filters included, but were not limited to, “epidemic”, “pandemic”, “COVID-19”, “virus”, “pneumonia”, “confirm”, “positive”, “lockdown”, “mask”, etc. In this paper, we quantified social media attention on day t as the addition of trending search counts from the two sources.

**Fig 4:**
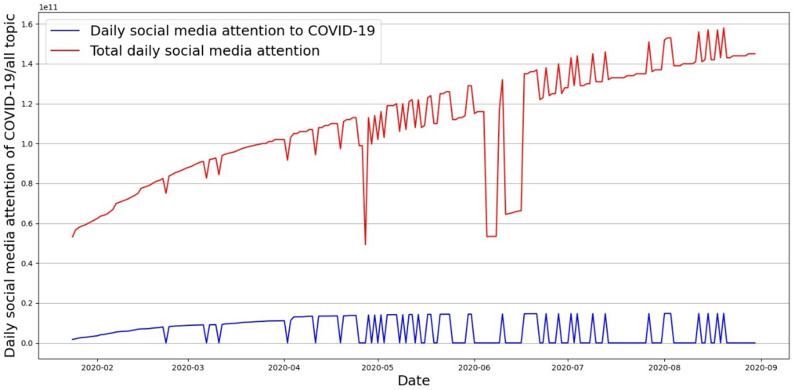
Social media attention of COVID-19 related vs. topics in total.

Fig 4 presents the comparison of social media attention to COVID-19-related topics and topics in total. It is interesting to find that public attention to the pandemic (blue line) always comes and goes quickly. The pandemic-related search counts are positively related to the total searches, meaning the outbreak of COVID-19, no matter on national or local scales, will make people more active on social media. However, it is also noticed that, as time went by, the proportion of people’s attention to the pandemic gradually decreased and stabilized at a low level.

### 3.2. Methods

To study the change in urban vehicle mobility and understand key factors during different periods of COVID-19, this paper proposes a sequential statistical modelling framework from the perspective of vehicle mobility loss. The framework consists of two components:

- A Prophet time series prediction model, which is used to predict vehicle mobility after January 20, 2020, under the assumption that there is no pandemic. In this way, daily vehicle mobility loss can be calculated using prediction value minus actual observation.
- A multiple linear regression model, which captures the impacts of influencing factors from both external (regulation orders) and inner (public fear) sides, using mobility loss as the dependent variable.

Prophet is a procedure for forecasting time series data based on an additive model where non-linear trends are fit with yearly, weekly, and daily seasonality, plus holiday effects [59]. Since being released by Facebook in 2017, it immediately became one of the most popular tools for predicting time series data. Although multiple pre-processing works have been done for the ANPR data, due to some inherent defects of the data acquisition devices, it is difficult to eliminate problems such as data missing and abnormality. Prophet works best with time series that have strong seasonal effects and several seasons of historical data, and it is robust to missing data and shifts in the trend and typically handles outliers well [60]. The reliable fitting ability of Prophet for traffic data processing and prediction is widely reported [59,61–63]. Therefore, the Prophet time series model is applied to the ANPR data here for predicting vehicle mobility trends if no pandemic happens.

A time series model can generally be defined by an observation equation that relates the observed time-series variables to a set of latent state variables, and a set of state equations which dictate how the latent state variables evolve over time under uncertainties [39]. In this paper, under the assumption that there is no COVID-19, the prediction value of vehicle mobility on day t (denoted as p(t)) is given by


p(t)=g(t)+s(t)+h(t)+ε(t) (3)

where g(t) is a trend function, it is a model for how vehicle mobility grows and how it is expected to continue growing. And g(t) can be expressed by a linear trend model


$$g(t)=(k+a(t{)}^{T}\delta )t+(m+a(t{)}^{T}\gamma )$$ (4)

where k denotes a varying growth rate of vehicle mobility and m is the offset parameter. a(t) is a vector of binary variables consisting of $a_{j}(t)$, which can be expressed as Equation (5). δ is a vector of the vehicle mobility change rate adjustments consisting of $\delta_{t}$, which can be expressed as Equation (6). And define $\delta \in {\mathbb{R}}^{J}$, in which suppose there are J changepoints and they are automatically selected given a set of candidates with the function in Equation (4) and by putting a Laplace prior on δ. γ is a vector of adjusted vehicle mobility change rates consisting of $\gamma_{j}$, which can be expressed as Equation (7). Suppose the jth changepoint is defined at day $t_{j}$, and defined $a_{j}(t)$ as one if day t is not before the changepoint j, otherwise define it as zero, as shown in Equation (5).


$$a_{j}(t)=\left\{ \begin{array}{c} 1,\;if\text{ t}\geq t_{j}\;\\ 0,\;otherwise \end{array}\right.\;$$ (5)

Let $\delta_{j}$ denote the vehicle mobility change in the rate at day $t_{j}$, then $\delta_{t}$ can be calculated as


$$\delta_{t}=k+\sum_{j:t>t_{j}}\delta_{j}$$ (6)

When vehicle mobility growth rate k is adjusted, the offset parameter m must also be synchronously adjusted to connect the endpoints of the segments. The adjusted vehicle mobility change rate at changepoint j can be expressed as


$$\gamma_{j}=(t_{j}-m-\sum_{l<j}\gamma_{l})(1-\frac{k+\sum_{l<j}\delta_{l}}{k+\sum_{l\leq j}\delta_{l}})$$ (7)

s(t) of Equation (3) denotes seasonal effects, which can be expressed by a standard Fourier series function as


$$s(t)=\sum_{n=1}^{N}\left(a_{n}\cos(\frac{2\pi nt}{P})+b_{n}\sin(\frac{2\pi nt}{P})\right)$$ (8)

where *P* = 365.25 represents the yearly seasonality and *N* = 10. h(t) of Equation (3) represents non-periodic irregular holiday effects, which can be expressed as


h(t)=Z(tkappa (9)

Where κ denotes the influence range of holidays. Z(t) is the indicator function, which can be expressed by a matrix of regressors as


$$Z(t)=[1(t\in D_{1}),...,1(t\in D_{L})]$$ (10)

ε(t) of Equation (3) represents a normally distributed error term, representing any idiosyncratic changes that are not accommodated by the model.

To evaluate model performance, weighted average absolute percentage error (WMAPE) is introduced to measure the prediction error, which can be expressed as


$$WMAPE=\frac{\sum_{t}\left|{\hat{r}}_{t}-p_{t}\right|}{\sum_{t}\left|r_{t}\right|}$$ (11)

Multiple linear regression is widely used to identify relationships, make predictions, and assess the influence of several variables on the outcome. It allows researchers to analyze how multiple factors, acting together, influence the dependent variable, while being simple in form, intuitive in results, and easy to understand. It is also computationally straightforward and can be easily implemented.

The objective of this research is to observe and analyze changes in overall urban vehicle mobility over time as the pandemic progresses, along with its main influencing factors. Combining literature analysis with available data resources, we use vehicle mobility loss as the dependent variable in the regression model, with indexes representing regulatory orders and public fear as independent variables. Vehicle mobility loss is defined as the difference between the predicted value assuming no pandemic happens and the observed real value after the pandemic occurs. Additionally, we hypothesize that regulatory orders and public fear either independently or interactively influence changes in vehicle mobility.

To capture the temporal dynamics of these influences, and inspired by Liu and Yamamoto [42], we introduce a variable called “time distance”, defined as the number of days from the start of outbreak day 0 to day t during a pandemic wave. The rationale for selecting time distance as a moderating variable lies in the observation that both public fear and the impact of policy strictness are not constant over time — their effects on behavior may diminish, accumulate, or fluctuate as the situation evolves. By including interaction terms between time distance and other independent variables, the model can better reflect how the joint effects of policy strictness and public fear on vehicle mobility change throughout the course of the pandemic.

Moreover, existing studies have shown that newly added confirmed cases can bring cumulative anxiety to individuals, implying a lag effect on perceived risk [37]. Therefore, lag terms for confirmed cases are also introduced. Interaction terms are constructed to acquire a deeper understanding of the relationships among these factors and how their combined influence evolves over time.

To fully investigate influencing factors in different stages of COVID-19, four regression models were built for the long term (i.e., the whole research period from January 21, 2020, to August 30, 2020) and three short-term periods (i.e., the national outbreak period, the stable transition period, and the regional outbreak period distinguished in Section 3). Ordinary least square estimation (OLS) is used to estimate the parameters. After multiple rounds of tests, the models were calibrated and the results can be found in Section 5.2. More details about the considered factors and the related terms can be found in S1 Appendix A and S2 Appendix B.

## 4. Results

### 4.1. Vehicle mobility loss

**Fig 5:**
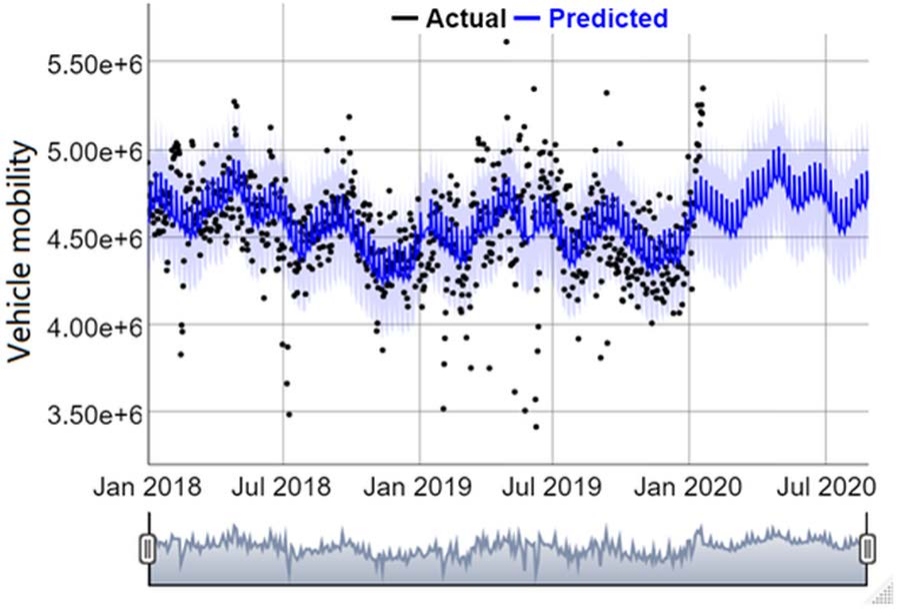
Vehicle mobility prediction result assuming there is no COVID-19 in 2020.

Following the modelling framework in Section 4, the Prophet time series model was used to predict vehicle mobility without COVID-19 by using data before the outbreak day (January 20, 2020). Fig 5 shows the prediction result, where the black dots are the actual observations, and the blue line is the predicted value with the light blue ribbon representing a 95% confidence interval. It can be seen that the prediction result fits satisfactorily and has been smoothed by the model.

To further evaluate model performance, WMAPE was calculated for 2019 (using data in 2018 as input to predict vehicle mobility in 2019) and for 2020 using Equation (8), respectively. The WMAPE 2019 is 0.00012 and WMAPE 2020 is 0.1572. Both WMAPE values are substantially smaller than 100%, indicating good fits of the Prophet time series model. A possible reason for the difference between WMAPE 2019 and WMAPE 2020 may be that mobility tends to be stable from 2018 to 2019 while the actual value in 2020 decreases largely due to the outbreak of COVID-19.

**Fig 6:**
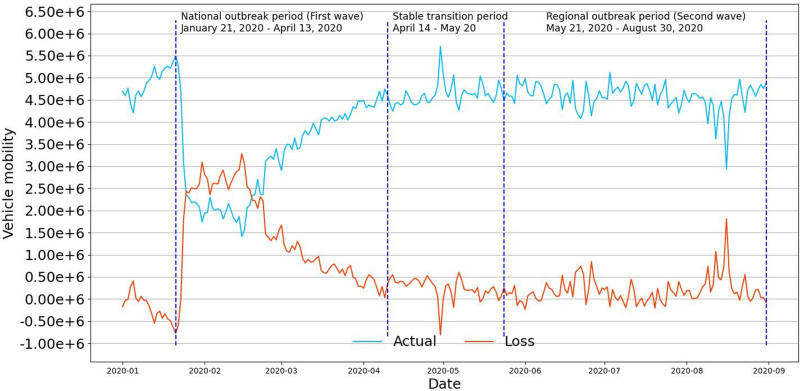
The actual, predicted, and loss of urban vehicle mobility for the study period.

Fig 6 visualizes the variation trends of actual vehicle mobility (cyan line) and the loss (yellow line). It is clear that the yellow line looks like a big inverted “V” during the national outbreak period (the first wave) and tends to be smoother later, indicating the loss in the first wave was much greater compared with the other two periods. It is also found that predicted vehicle mobility largely overlaps with the actual observation in the stable transition period and the regional outbreak period (the second wave), indicating a rapid recovery in mobility once the pandemic gets well controlled.

Moreover, to quantify vehicle mobility changes under COVID-19, the loss was divided by the predicted mobility, turning out that Mianyang city lost 29.66% in vehicle mobility during the national outbreak period, 5.04% during the stable transition period, and 3.97% during the regional outbreak period. Vehicle mobility significantly decreased in the first wave. With the stabilization of the situation, vehicle mobility gradually recovered to the level before the outbreak of the pandemic and was no longer affected by the subsequent outbreak of the pandemic. The reasons for this trend will be analyzed in the following section, combining the results from the regression model.

### 4.2. Multiple linear regression results

Based on the quantitative result from Section 4.1, nearly 30% loss in vehicle mobility was observed during the first wave and only minor losses were captured in the following time even though sporadic pandemic still occurred. To deeply explore the drivers of this trend, four multiple linear regression models were built as mentioned in Section 3.2, named MODEL 1, MODEL 2, MODEL 3, and MODEL 4. These models were tested for the efficacy and robustness of the variables. Table 1 shows the calibrated results of the models.

**Table 1:** Multiple linear regression analysis results for vehicle mobility loss in different periods.

Model /Independent variable	MODEL 1 (Whole study period)	MODEL 2 (National outbreak period)	MODEL 3 (Stable transition period)	MODEL 4 (Regional outbreak period)
Coefficient	Coefficient	Coefficient	Coefficient
Constant	571652***	671921***	149142.800**	143774.660*
Interaction variable	79491494***	76065360***	−9692.000	−9.817
Economy support index	−430479***	−678970	NA	NA
China confirmed cases in the last seven days (lag variable)	1886321***	1772636***	404.800**	125.563
Number of observations	219	80	37	102
R^2^	0.814	0.795	0.180	0.030
Adjusted R^2^	0.812	0.787	0.133	0.011
F	315.000***	98.480***	3.835**	1.548
AIC	6238.315	2319.242	1050.045	2861.878

*Note:* Interaction variable = Confirmed cases in Sichuan × Media attention × Stringency index/ time distance, *Coefficient significant at p < 0.10, **Coefficient significant at p < 0.05, ***Coefficient significant at p < 0.001.

Table 1 suggests a high statistical fit for MODELs 1 and 2 with adjusted R^2^ of 0.812 and 0.787, respectively. However, the adjusted R^2^ values for MODELs 3 and 4 are 0.133 and 0.011 respectively, indicating that the assumed independent variables are unable to explain vehicle mobility loss for the stable transition and regional outbreak period. Thus, the following analysis is mainly focused on the results of MODEL 1 and MODEL 2. Let $y_{WP}$ denote mobility loss during the whole study period and $y_{NOP}$ denote the mobility loss during the national outbreak period. The specific form of MODEL 1 and MODEL 2 can be written as


$$y_{WP}=571652+79491494\times \frac{x_{ConSC}\times x_{MA}\times x_{SI}}{X_{TD}}-430479\times x_{ES}+1886321\times x_{ConC7},$$ (12)

$$y_{NOP}=671921+76065360\times \frac{x_{ConSC}\times x_{MA}\times x_{SI}}{X_{TD}}+1772636\times x_{ConC7}.$$ (13)

where $x_{ConSC}$ is the confirmed cases in Sichuan; $x_{MA}$ is the media attention index (represented as trending searches as described in Section 3.1); $x_{SI}$ is the stringency index; $X_{TD}$ is the time distance as defined in Section 3.2. $x_{ES}$ is the economy support index, and $x_{ConC7}$ is the lag variable, which represents China confirmed cases in the last seven days.

Before presenting this result, we tried multiple ways of regression (see S1 Appendix A). Unfortunately, the fittings of regression models for the stable transition period and the regional outbreak period cannot be able to reach satisfactory values with our data. A possible explanation is that for the two periods, the impact of COVID-19 on vehicle mobility loss is not significant, and the losses are more likely to be related to the random changes of urban travel demand. To verify this assumption, the ANPR data needs to be re-preprocessed. However, this deviates from the research objective of this paper. Thus, we leave it to the future work.

**Fig 7:**
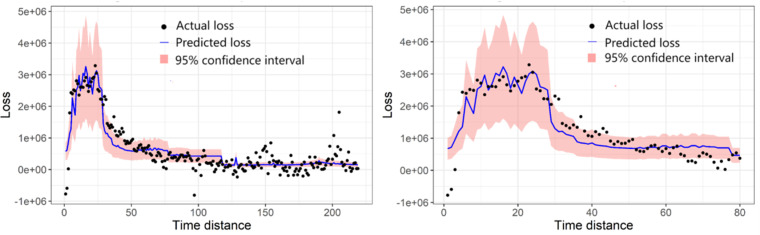
Regression fit of vehicle mobility loss.

Fig 7 visualizes the regression fit of vehicle mobility loss for MODEL 1 and MODEL 2, respectively, In the figure, the black dots represent the actual loss value, and the blue line with the red ribbon represents the predicted loss and its 95% confidence interval. It can be observed that the regression model performs satisfactorily in the early stage from Fig 7b; however, after the first wave, the deviation becomes larger and larger as shown in Fig 7a.

The signs and the significance levels of the parameters presented in Table 1 identify the relationship between vehicle mobility loss and the explanatory variables. The economy support index and the lag variable (China confirmed cases in the last seven days) are both independently significant. The positive signs of the lag variable in MODEL 1 and MODEL 2 verify our assumption that the number of nationwide confirmed cases has a negative lagging effect on the travel intensity of local residents, and the lag time is around seven days. Moreover, it is interesting to see that the economy support index is not significant during the national outbreak period (MODEL 2) yet negative for the whole research period (MODEL 1). This is mainly because all parts of China began to implement relevant policies focusing on encouraging economic recovery at the end of the national outbreak period, as the cyan line shown in Fig 2. It means that it takes a while for the economic policies to come into play, suggesting that a lag effect may also exist for this variable.

A more important finding of our research lies in the interaction variable, which consists of confirmed cases in Sichuan province, social media attention, stringency index, and time distance. It is strongly significant in both MODEL 1 and MODEL 2, suggesting that during the entire research period, especially during the national outbreak period, vehicle mobility loss was primarily due to the joint effects of these factors. This finding is consistent with the conclusions of a study by Sung [64] on vehicle mobility in Seoul during the COVID-19 pandemic. They found that relying solely on regulatory orders to restrict vehicle mobility had limited effectiveness, and that policy interventions should take more complex risk-avoidance behaviors into consideration.

**Fig 8:**
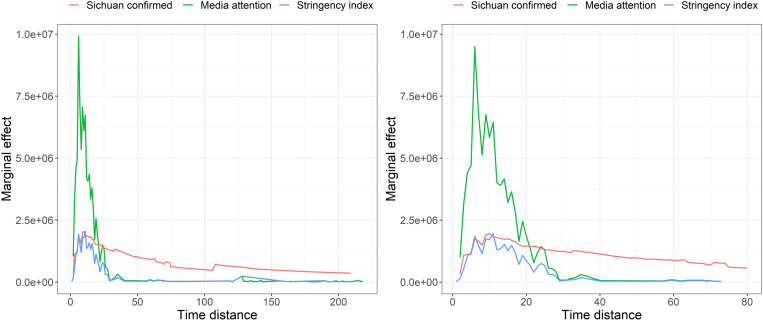
The change of marginal effects on vehicle mobility loss over time.

To further understand how public fear (represented as social media attention and confirmed cases) and policy strictness interact with each other, the marginal effect of the interaction term is obtained by calculating its partial differential of the corresponding variable. Fig 8 presents the result and the effect changes with time in MODEL 1 and MODEL 2.

The marginal effects of these variables on vehicle mobility loss initially increase and then decrease, which is consistent with the general pattern of changes in marginal effects. It indicates that people feel not motivated to completely follow the Prevention Guidelines with the continuous recurrence of the pandemic, a phenomenon that has been largely verified and known as “caution fatigue” [39,28,29]. Our study goes a step further than previous research by quantifying the potential timing of caution fatigue through marginal effects analysis. Fig 8 shows that people’s “freshness” to COVID-19 (actively paying attention to relevant information or actively responding to various policies) can last for around 30 days. Subsequent facts also show that caution fatigue makes the control policies of many countries very difficult to implement in the later stage of the pandemic (especially in 2022). Some Asian countries, such as Vietnam and China, which have achieved ideal pandemic prevention performance by relying on dynamic clearing in the early stage, have also been almost forced to open up in the later stage when the conflict between people’s willingness to resume normal life and “clearing” is irreconcilable.

Although the overall trend of the impact of policy control (represented by stringency index) and public fear (represented by confirmed cases and social media attention) on vehicle mobility loss is consistent, as shown in Fig 8, there is a significant difference in the magnitude of their effects. Seeing from the marginal effect of social media attention (the green line), it reaches a peak in a short time (around 10 days after the outbreak of COVID-19) and declines very fast. This indicates that the perceived risk will rapidly accumulate, and people will generally magnify this threat, thus largely altering their short-term travel decisions. However, this effect comes and goes quickly. The public soon accepts the status quo and becomes numb. It is interesting to note that when China’s macro anti-pandemic policy has begun to shift from “dynamic clearing” to “coexistence with the virus”, the status of social media in China and people’s reactions also confirm the characteristic of caution fatigue of media attention. The marginal effect trend of the stringency index (the blue line) is similar to media attention, yet the magnitude of its impact on mobility loss is much smaller. Compared with social media attention and stringency index, the impact of confirmed cases is more moderate, but this impact is more stable and sustained.

## 5. Discussion

**Fig 9:**
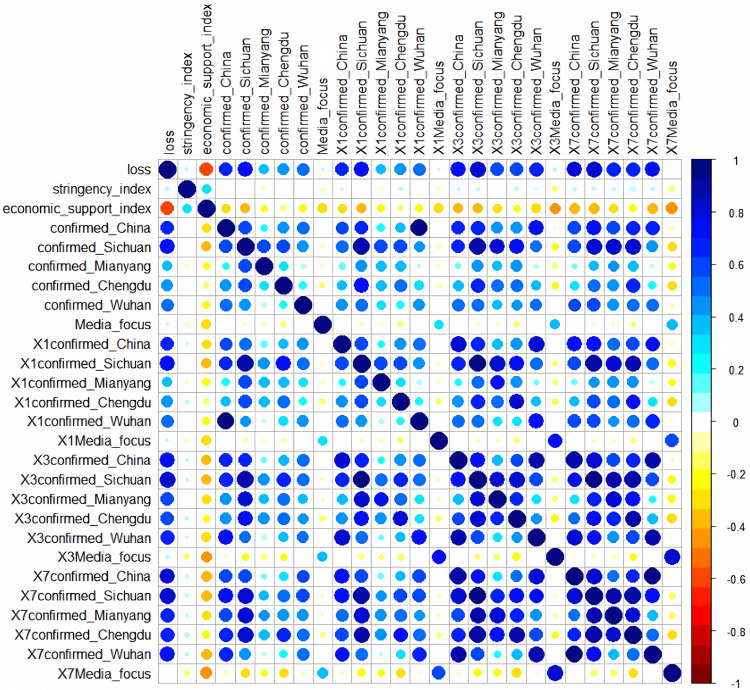
Correlation analysis of initial explanatory variables results.

**Fig 10:**
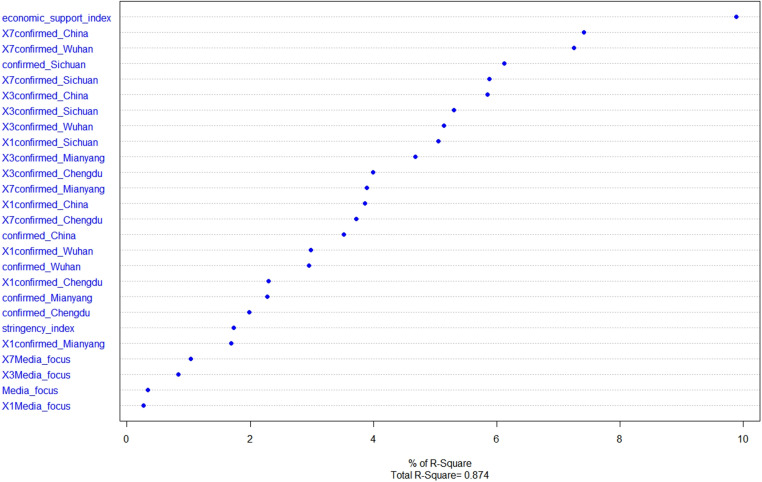
Variable importance ranking result.

This section focuses on the specific process of conducting regression in this empirical study and discusses the transferability of the method.  The factors in the regression models were determined by carefully considering the results of multiple selection analysis steps. For each regression model, it started with all possible explanatory variables, and then considered how deleting a variable affects either the Akaike information criterion (AIC) or the Bayesian information criterion (BIC). A backward search was performed on the explanatory variables under both criteria, and the model with the smallest leave-one-out cross-validation (LOOCV) root mean square error was selected in the end. As for the initial factors, five spatial variables were introduced at the beginning, which are confirmed cases in Mianyang city, Chengdu city, Wuhan city, Sichuan province, and China, to observe the spatial impact of confirmed cases in different distance scales on local vehicle mobility. Among them, Mianyang is the background city, while Chengdu is the capital city of Sichuan province and the nearest metropolitan area to Mianyang. Wuhan is the capital city of Hubei province and the first city in China to break out during the COVID-19 pandemic. Moreover, lagging variables, which represent the time lag effect of risk perception, were also introduced. Terms denoting one-day lag, three-day lag and seven-day lag, respectively, were created for the five confirmed cases variables and the media attention index. Introducing so many related variables might cause severe multicollinearity problems. To overcome this issue, stepwise regression and full subset regression were used separately with the initial explanatory variables (see S1 Appendix A for detailed results). Furthermore, a manual selection was conducted based on the results of correlation analysis and variable importance ranking, as shown in Figs 9 and 10. The correlation analysis can help identify the main source of multicollinearity, while the variable importance ranking can help us keep the collinear variables with higher importance.

Finally, a high goodness of fits with few key explanatory variables and a satisfactory refinement of the model is achieved.

The multiple linear regression model proposed in this paper is simple in form. However, we believe the ultimate goal of modelling is to solve a problem rather than presenting a fancy method [65]. Following the principle of keeping simplicity, interpretability, and high goodness of fits, the final form of the model is chosen from a pretty vast of tested models (some of the alternatives can be found in S2 Appendix B). We did not think of interacting risk perception (i.e., media attention and confirmed cases), policy control (i.e., stringency index), and time distance at the beginning. This rule was found by observing the correlation of variables and testing a large number of interactive terms. The final model is more concise without losing its good performance. In the meanwhile, the final model is capable of explaining the interactive effect between risk perception and policy control.

We believe the proposed method, including how to select the key factors and the final form of the regression model, has good transferability for two reasons. First, in the era of big data, the essence of data analysis is usually not how to obtain multi-dimensional massive data, but to identify which data is dominant. We adopt a multi-step method, which consists of stepwise/ full subset regression (either one of them is suggested for readers who would like to try variable selection work) and manually selecting based on correlation analysis and variable importance ranking. From the results shown in Table 1, the key factors all have strong statistical significance. Second, the regression model is generally used as the base of attribution analysis. It has some fascinating features, such as being easy to understand and use, clearness in interpretation, etc. As mentioned in Section 5, the interaction term is determined after multiple rounds of tests. And we would encourage our readers to do so to explore the inner rules of their data. Of course, there are still many machine learning methods that can further improve the accuracy of the model prediction. It will be a future direction, but we suggest modelers keep a good balance between the goodness of fits and the interpretability of the method.

## 6. Conclusions

This paper investigates how and why urban overall vehicle mobility changes in the first eight months when COVID-19 outbreaks. Three steps were employed to capture the variations in vehicle mobility over time and the driving factors. First, a large multisource dataset was built from three data sources. The ANPR data that represents changes in vehicle mobility from January 2018 to August 2020 was used, and data that represents the main factors from January to August 2020 were crawled, making the total number of raw data for processing exceeded 4.3 billion records. With this big data, what kind of precipitous decline in vehicle mobility and how to recover slowly in the next few months can be intuitively observed (Fig 2). Second, the Prophet time series model was applied to predict vehicle mobility without COVID-19 and the loss can be obtained by subtracting the real value from the predicted value. Third, multiple linear regression was applied to fit the loss. Factors were carefully selected by considering the results of stepwise regression, full subset regression, correlation analysis, and variable importance ranking (see S1 Appendix A). The specific form of linear regression, especially the construction of the interaction term, was also determined through meticulous design and multiple rounds of testing (see S2 Appendix B). By doing so, two models with high goodness of fits and concise in forms for different pandemic periods were built, see equations (12) and (13).

The research findings have important implications for transportation authorities. First, the analysis of vehicle mobility shows that urban mobility loss during the national outbreak period was 29.66%, while losses during the stable transition and regional outbreak periods were 5.04% and 3.97%, respectively. This indicates that, despite a second wave in some areas, vehicle mobility generally returned to pre-pandemic levels. Second, both regulatory orders and public fear contributed to mobility loss, but in different ways. Economic support positively impacted mobility, while policy stringency interacted with public fear to affect mobility. Early in the pandemic, public reactions to control policies were similar to media-perceived risks, with caution fatigue emerging later. Public fear had a stronger short-term impact, but its effect on reducing travel plans persisted in the long term. Lastly, this study underscores the importance of considering caution fatigue when formulating policies. As fear diminishes over time, policymakers face greater challenges in managing the later stages of a pandemic. When strict lockdowns end, alternative strategies, such as remote work, may become less effective, requiring a combination of interventions. We believe the empirical results obtained from this study are of great practical value. For other researchers, who are interested in the impact of the pandemic on their own city traffic but lack data at hand, our quantitative results on vehicle mobility loss can provide them with a reference for evaluation.

The findings of this study may still be applicable to other cities with similar urban structures and transportation systems, especially in the context of global health crises or other large-scale disruptions. However, the study’s applicability may vary depending on factors such as city size, transportation infrastructure, and local policies. In the future, we plan to explore how this model can be adapted to different regions, considering local socioeconomic conditions and the evolution of transportation behavior over time. Additionally, besides analyzing the impact of various factors on vehicle mobility during the COVID-19 pandemic, this paper also presents a paradigm for conducting quantitative and in-depth analysis of travel behavior by integrating multi-source, massive, and heterogeneous big data. This approach not only applies to the study of transportation behavior during “black swan” events like the COVID-19 pandemic but can also be extended to explore how transportation behavior changes under the influence of disruptive new policies or significant shifts in external environments.

This study has several limitations. First, the analysis was conducted at a macro level based on ANPR data, which lacks socio-demographic attributes. As a result, it cannot capture how mobility changes differ among population groups, such as by age, income, or occupation. Future research could address this by integrating household travel surveys, mobile phone signaling data, or classifying vehicles into categories such as private cars, taxis, online hailing vehicles, and buses. Second, while this study uses time distance to reflect the temporal moderating effect in the analysis, other potential moderating factors could be considered in future research. For example, moderators such as geographical distance, media exposure, socioeconomic status, or policy type might provide additional insights into how different regions or population groups respond to pandemic-related restrictions and public fear. Including such factors could help reveal more nuanced interaction effects between external influences and mobility behavior changes. Third, the ANPR data did not include details about trip purposes, travel time, or distance — important indicators for understanding changes in travel behavior during a pandemic. Future studies could focus on extracting these characteristics to conduct more comprehensive and behaviorally meaningful analyses. Lastly, the study period was limited to the first eight months after the COVID-19 outbreak due to data availability constraints. Yet, the pandemic and its associated policy measures continued to evolve. Extending the research period and expanding the scope to cover multiple cities or countries would help assess the consistency and generalizability of the findings. Furthermore, incorporating recent studies, such as the investigation into labor supply decisions of taxi drivers in megacities during the pandemic, could complement the current work by offering insights from both travel demand and service supply perspectives.

## Supporting information

S1 Appendix A. Determination of the factors. (DOCX)

S2 Appendix B. Determination of the linear regression model. (DOCX)
