# Supplementary material for: How regulatory orders and public fear affect vehicle mobility under COVID-19: A global perspective from urban overall vehicles using multi-source data
Source: PLoS One. 2025 Jun 11;20(6):e0325118. doi: 10.1371/journal.pone.0325118 (PMC12157307; doi:10.1371/journal.pone.0325118)
Supplement: S1 Appendix — (DOCX) [file pone.0325118.s001.docx]

**Appendix A Determination of the factors**

In the beginning, all 26 variables were considered in the linear regression and were tested by variance expansion factor (VIF). Many variables are proven to have serious multicollinearity problems. The test results can be found in [Table A.1](#tabA1).

**Table A1.** Multiple linear regression results with initial explanatory variables.

| **Variable** | **Estimate** | **Std. Error** | **t value** | **Pr(>\|t\|)** | **VIF** |
| --- | --- | --- | --- | --- | --- |
| Intercept | -4.510E+05* | 1.890E+05 | -2.390 | 0.018 | - |
| Stringency_index | 1.380E+04*** | 2.340E+03 | 5.910 | 0.000 | 1.223 |
| Economic_support_index | -1.230E+04*** | 2.010E+03 | -6.080 | 0.000 | 2.617 |
| Confirmed_China | 3.330E+02 | 2.800E+02 | 1.180 | 0.236 | 269.662 |
| Confirmed_Sichuan | 3.710E+04** | 1.250E+04 | 2.970 | 0.003 | 10.508 |
| Confirmed_Mianyang | 1.340E+05 | 7.700E+04 | 1.740 | 0.083 | 2.709 |
| Confirmed_Chengdu | 6.180E+03 | 2.860E+04 | 0.216 | 0.829 | 7.229 |
| Confirmed_Wuhan | 7.820E-01 | 3.130E+01 | 0.025 | 0.980 | 2.193 |
| Media_focus | -2.260E+01 | 4.030E+01 | -0.561 | 0.576 | 1.279 |
| X1Confirmed_China | 2.940E+00 | 4.260E+01 | 0.069 | 0.945 | 6.227 |
| X1Confirmed_Sichuan | -9.840E+02 | 2.600E+04 | -0.038 | 0.970 | 45.653 |
| X1Confirmed_Mianyang | -2.660E+05** | 8.120E+04 | -3.275 | 0.001 | 3.070 |
| X1Confirmed_Chengdu | -3.070E+04 | 2.210E+04 | -1.393 | 0.165 | 4.300 |
| X1Confirmed_Wuhan | -4.140E+02 | 2.980E+02 | -1.386 | 0.167 | 199.306 |
| X1Media_focus | -4.880E+01 | 5.200E+01 | -0.939 | 0.349 | 2.128 |
| X3Confirmed_China | -3.100E+01 | 3.450E+01 | -0.900 | 0.369 | 25.720 |
| X3Confirmed_Sichuan | -3.480E+03 | 1.440E+04 | -0.242 | 0.809 | 117.243 |
| X3Confirmed_Mianyang | 3.960E+05*** | 9.390E+04 | 4.219 | 0.000 | 16.733 |
| X3Confirmed_Chengdu | 5.260E+04*** | 1.450E+04 | 3.626 | 0.000 | 10.984 |
| X3Confirmed_Wuhan | 8.680E+01* | 4.060E+01 | 2.137 | 0.034 | 20.306 |
| X3Media_focus | 6.210E+01 | 3.460E+01 | 1.797 | 0.074 | 4.360 |
| X7Confirmed_China | -2.330E+00 | 2.680E+01 | -0.087 | 0.931 | 70.397 |
| X7Confirmed_Sichuan | 1.720E+04** | 6.090E+03 | 2.820 | 0.005 | 107.469 |
| X7Confirmed_Mianyang | -2.270E+05*** | 5.050E+04 | -4.485 | 0.000 | 21.220 |
| X7Confirmed_Chengdu | -4.460E+04*** | 9.900E+03 | -4.499 | 0.000 | 20.690 |
| X7Confirmed_Wuhan | 3.760E+01 | 2.800E+01 | 1.343 | 0.181 | 37.667 |
| X7Media_focus | -1.020E+01 | 1.740E+01 | -0.585 | 0.559 | 4.030 |
| Multiple R^2^ | 0.874 |  |  |  |  |
| Adjusted R^2^ | 0.857 |  |  |  |  |

VIF>4 indicates that a multicollinearity problem exists, while VIF>10 means the problem is very serious

Step forward and backward regression is used in this paper. The model will add a variable each time and make an evaluation. Variables that do not contribute to the model will be deleted. The prediction variables may be added and deleted several times until the optimal model is obtained. After applying the stepwise regression method, VIF was used again for testing (see [Table A.2](#tabA2) for regression and test results). We found that although some variables were deleted, there was still a serious multicollinearity problem. Therefore, it is impossible to get the final scheme of variable selection only by one-step regression.

**Table A.2** Stepwise regression and test result.

| **Variable** | **Estimate** | **Std. Error** | **t value** | **Pr(>\|t\|)** | **VIF** |
| --- | --- | --- | --- | --- | --- |
| Intercept | -4.818E+05** | 1.716E+05 | -2.808 | 0.005 | - |
| Stringency_index | 1.370E+04*** | 2.280E+03 | 6.008 | 0.000 | 1.205 |
| Economic_support_index | -1.171E+04*** | 1.774E+03 | -6.600 | 0.000 | 2.11 |
| Confirmed_China | 3.129E+02 | 2.213E+02 | 1.414 | 0.159 | 174.641 |
| Confirmed_Sichuan | 3.454E+04** | 1.082E+04 | 3.192 | 0.002 | 8.173 |
| Confirmed_Mianyang | 1.510E+05* | 6.696E+04 | 2.255 | 0.025 | 2.124 |
| X1Confirmed_Mianyang | -2.704E+05*** | 7.893E+04 | -3.426 | 0.001 | 3.004 |
| X1Confirmed_Chengdu | -3.096E+04 | 1.933E+04 | -1.602 | 0.111 | 3.423 |
| X1Confirmed_Wuhan | -3.574E+02 | 2.414E+02 | -1.480 | 0.140 | 135.258 |
| X3Confirmed_Mianyang | 4.059E+05*** | 7.217E+04 | 5.625 | 0.000 | 10.244 |
| X3Confirmed_Chengdu | 4.998E+04*** | 1.205E+04 | 4.150 | 0.000 | 7.864 |
| X3Confirmed_Wuhan | 6.160E+01** | 2.256E+01 | 2.731 | 0.007 | 6.490 |
| X3Media_focus | 2.922E+01 | 2.002E+01 | 1.459 | 0.146 | 1.515 |
| X7Confirmed_Sichuan | 1.568E+04*** | 3.654E+03 | 4.291 | 0.000 | 40.162 |
| X7Confirmed_Mianyang | -2.401E+05*** | 4.410E+04 | -5.445 | 0.000 | 16.765 |
| X7Confirmed_Chengdu | -4.272E+04*** | 8.660E+03 | -4.933 | 0.000 | 16.409 |
| X7Confirmed_Wuhan | 2.738E+01 | 1.503E+01 | 1.822 | 0.070 | 11.270 |
| Adjusted R^2^ | 0.862 | | | | |

Full subset regression means that all possible models will be tested. The ordinate of the full subset regression diagram (as shown in [Figure A.1](#figA1)) represents the adjusted R square, and the small black block in the row of each R square represents the variables selected by the model at the R square level. For example, when the R square is 0.78, the selected model variables are economic support, the 7-day lag term of the number of confirmed cases in Sichuan, and the intercept term. It can be seen from the figure that there are many variable schemes. Eliminating multicollinearity is not the only purpose of selecting model variables, but also needs to consider the fitting performance, interpretation ability, and simplicity of the model.


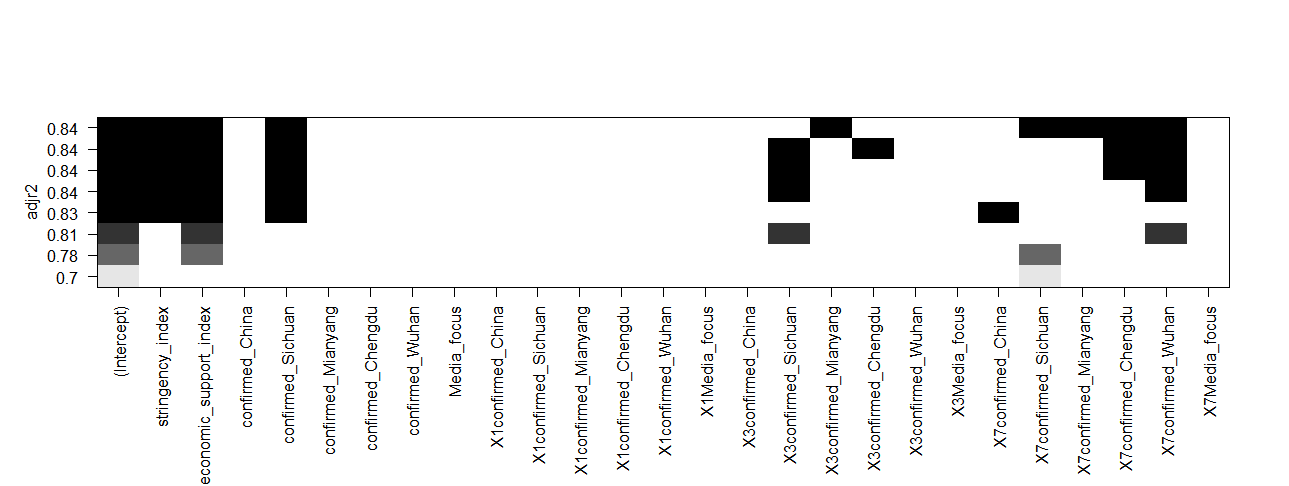


**Figure. A.1.** Full subset regression diagram.
