# Supplementary material for: How regulatory orders and public fear affect vehicle mobility under COVID-19: A global perspective from urban overall vehicles using multi-source data
Source: PLoS One. 2025 Jun 11;20(6):e0325118. doi: 10.1371/journal.pone.0325118 (PMC12157307; doi:10.1371/journal.pone.0325118)
Supplement: S2 Appendix — (DOCX) [file pone.0325118.s002.docx]

**Appendix B Determination of the linear regression model**

To keep the length of this paper at a reader-friendly level, only a small part of the tested models are listed in [Table B.1](#tabB1). In the process of determining the final model, we are far from debugging these models. It is a cyclical process from selecting variables, determining interaction items, establishing models, diagnosing models to finally determining models.

**Table B.1** Part of the tested models and results

|  | **Interaction variable** |  |  |  |
| --- | --- | --- | --- | --- |
| **Model 1** | $(\text{Confirme}\text{d}_{\text{7China}}\times\text{Media attentio}\text{n}_{7})\text{/}\text{d}_{\text{time}}$ | | | |
| **Model 2** | $(\text{Media attentio}\text{n}^{\text{0.8}}\times\text{confirme}\text{d}_{\text{Sichuan}}^{\text{0.12}})/d_{\text{time}}$ | | | |
| **Model 3** | $(\text{Media attention}\times\text{confirme}\text{d}_{\text{Sichuan}})/d_{\text{time}}$ | | | |
| **Variables of Model 1** | Estimate | Std. Error | t value | Pr(>\|t\|) |
| Intercept | -7.076E+05** | 2.662E+05 | -2.658 | 8.460E-03 |
| Interaction variable | 4.795E+01*** | 3.002E+00 | 15.970 | <2E-16 |
| Stringency_index | 1.253E+04*** | 3.271E+03 | 3.829 | 1.690E-04 |
| Economic_support_index | -1.525E+04*** | 1.875E+03 | -8.130 | 3.550E-14 |
| Confirmed_Sichuan | 3.030E+05*** | 6.172E+04 | 4.909 | 1.820E-06 |
| Media attention | 2.378E+05*** | 6.247E+04 | 3.807 | 1.840E-04 |
| Confirmed_China | -7.164E+03*** | 5.506E+03 | -1.301 | 1.946E-01 |
| Multiple R^2^ | 0.800 |  |  |  |
| AIC | 6254.938 |  |  |  |
| **Variables of Model 2** | Estimate | Std. Error | t value | Pr(>\|t\|) |
| Intercept | -1.346E+06*** | 1.863E+05 | -7.227 | 8.870E-12 |
| Interaction variable | 4.001E+04*** | 8.423E+03 | 4.750 | 3.750E-06 |
| Stringency_index | 1.234E+04*** | 2.600E+03 | 4.747 | 3.800E-06 |
| Economic_support_index | -9.443E+03*** | 1.698E+03 | -5.563 | 8.000E-08 |
| Confirmed_Mianyang | 1.504E+05*** | 7.743E+04 | 1.943 | 5.340E-02 |
| X7confirmed_Mianyang | -1.386E+03*** | 3.044E+04 | -0.046 | 9.637E-01 |
| X3confirmed_Mianyang | 9.555E+04*** | 5.824E+04 | 1.641 | 1.024E-01 |
| X7confirmed_China^0.2 | 3.068E+05*** | 2.415E+04 | 12.702 | <2E-16 |
| Multiple R^2^ | 0.838 |  |  |  |
| AIC | 6209.636 |  |  |  |
| **Variables of Model 3** | Estimate | Std. Error | t value | Pr(>\|t\|) |
| Intercept | -1.217E+06*** | 1.654E+05 | -7.358 | 3.990E-12 |
| Interaction variable | 1.648E+03*** | 1.912E+02 | 8.615 | 1.580E-15 |
| Stringency_index | 1.185E+04*** | 2.337E+03 | 5.071 | 8.590E-07 |
| Economic_support_index | -1.074E+04*** | 1.563E+03 | -6.874 | 6.800E-11 |
| X3confirmed_Mianyang | -2.427E+04*** | 3.878E+04 | -0.626 | 5.320E-01 |
| X7comfirm_China^0.2 | 2.931E+05*** | 2.006E+04 | 14.615 | <2e-16 |
| Multiple R^2^ | 0.858 |  |  |  |
| AIC | 6179.175 |  |  |  |
